# Beyond Biocontrol: Species‐Dependent Growth Promotion of Forage Grasses by *Metarhizium anisopliae* Isolates

**DOI:** 10.1002/jobm.70211

**Published:** 2026-10-27

**Authors:** Dylan Thomas Telles Amandio, Alex Sandro Poltronieri, Michele Trombin de Souza, Leandro do Prado Ribeiro

**Affiliations:** ^1^ Federal University of Santa Catarina (UFSC) Florianópolis Santa Catarina Brazil; ^2^ Research Center for Family Agriculture Agricultural Research and Rural Extension Company of Santa Catarina Research (CEPAF/EPAGRI) Chapecó Santa Catarina Brazil

**Keywords:** biological inoculants, endophytic entomopathogens, growth promotion, pastures

## Abstract

Research on the interaction between entomopathogenic fungi used in biological control programs and forage grass species remains limited. This study evaluated the effects of inoculating seedlings of the forage grass species *Axonopus catharinensis* cv. SCS315 Catarina Gigante, *Hemarthria altissima* cv. Florida, and *Cynodon dactylon* cv. Tifton 85 with five native and two commercial isolates of *Metarhizium anisopliae*. Plant growth responses varied according to both fungal isolate and forage species. In *C. dactylon* and *A. catharinensis*, specific isolates significantly increased leaf and root fresh and dry biomass, leaf number, and plant height. In contrast, similar positive effects were generally not observed in *H. altissima*, in which some isolates even reduced certain growth parameters. Considering overall plant performance (shoot and root traits combined), isolates CEPAF_ENT25, CEPAF_ENT27, and IBCB425 were particularly effective in promoting growth of *A. catharinensis* and *C. dactylon*. Root inoculation successfully established a fungal‐plant interaction, as *Metarhizium* spp. were recovered from all evaluated plant tissues (roots, stems, and leaves), with higher colonization frequencies in roots. Although all isolates were reisolated from the substrate, recovery rates decreased over time. These findings demonstrate substantial variability in plant growth‐promoting effects among *M. anisopliae* isolates and differential responsiveness among forage species. The results highlight the potential of selected isolates as multifunctional biological agents for sustainable pasture systems.

## 1 Introduction

The southern region of Brazil is dominated by small family farms focused on milk and meat production [1, 2], often using perennial pastures [3]. The livestock sector in Brazil relies heavily on natural and cultivated pastures, which cover approximately 177 million hectares [4], and is recognized for its cost efficiency [5] and high profitability [3]. Perennial grasses (Poaceae) are among the most widely cultivated forage crops in the Pantropical region, prized for their diverse improved cultivars, adaptability to various conditions, and high biomass production [6]. Common species in southern Brazil's perennial pasture systems include giant missionary grass (*Axonopus catharinensis*), hemarthria (*Hemarthria altissima*), and bermudagrass (*Cynodon dactylon*) [7, 8]. Nevertheless, ongoing innovation and technological advances are essential for creating sustainable systems and addressing challenges in extensive animal production and agropastoral management [9].

Pasture productivity can be limited by arthropod pest infestations, including the spittlebug complex (Hemiptera: Cercopidae), which reduce forage yield and nutritional quality by feeding on grasses and causing chlorosis, necrosis, and tiller death [10, 11, 12]. Although the bioecological aspects of this spittlebug complex remain poorly understood [13], severe infestations can cause productivity losses of up to 80% [11]. Entomopathogenic fungi, particularly *Metarhizium anisopliae* (Metsch.) Sorokin (Hypocreales: Clavicipitaceae), are widely used as biological control agents in these production systems [14, 15, 16, 17, 18, 19] and their use has increased with the growing availability of commercial formulations registered in Brazil [17].

Beyond pest suppression, *M. anisopliae* has been recognized as a multifunctional plant‐associated microorganism [20, 21]. This occurs through a protocooperative association as these fungi colonize the rhizosphere and establish endophytic relationships, promoting growth and improving nutritional quality [15, 16]. Endophytic colonization can be systemic or localized within inoculated plants [22, 23] and can be established through seed or root inoculation [24] as well as foliar spraying [25]. Root and endophytic associations can influence plant growth, nutrient acquisition, and photosynthetic isolates on the growth parameters, as well as tolerance to biotic and abiotic stressors [26, 27, 28]. For example, seed inoculation with *M. robertsii* resulted in systemic colonization and increased plant height and aboveground biomass in maize [24, 29], while similar growth‐promoting effects have been reported in maize treated with *M. anisopliae* [30, 31]. In cover crops, seed inoculation with *M. robertsii* resulted in fungal recovery from Austrian winter pea, cereal rye and winter canola, and significantly increased plant height and aboveground biomass in Austrian winter pea and cereal rye, but not in canola, highlighting the host‐dependent nature of these growth‐promoting responses [24]. Similar growth‐promoting effects have been reported in wheat colonized by *Metarhizium brunneum* (Petch) (Hypocreales: Clavicipitaceae) [32], supporting the broader potential of *Metarhizium* as a plant‐associated growth promoter. *Metarhizium* can also transfer insect‐derived nitrogen from cadavers to plants, influence root development through auxin‐related mechanisms, and modulate plant defense responses [33, 34]. Additionally, *M. anisopliae* affects the plants’ production of photosynthetic pigments [30] and nutrient [15, 16, 23], thereby enhancing the nutrition and growth of these forage species. Moreover, the establishment and functional outcome of *Metarhizium*‐plant associations are not uniform. These interactions are dependent on fungal isolate, host plant species, inoculation method, substrate conditions, and the surrounding microbial community [35, 36, 37, 38].

Although previous studies have highlighted the plant‐associated benefits of *Metarhizium*, no research has examined the effects of endophytic colonization by native *M. anisopliae* isolates on key forage grasses cultivated in southern Brazil, including *A. catharinensis*, *H. altissima*, and *C. dactylon*. In this sense, this study hypothesizes that these native isolates will establish endophytically in forage grasses and enhance plant vigor and biomass. We also predict that endophytic interactions will vary depending on entomopathogenic fungal isolate and host species. To address these hypotheses, we assessed the growth‐promoting effects of five native *M. anisopliae* isolates from the pastoral areas of Santa Catarina, Brazil, and two commercial isolates formulated into mycoinsecticides, using seedling transplants—the most commonly used method for establishing these forage species. We also evaluated the isolates’ effects on photosynthetic pigment levels across host species and confirmed endophytic colonization in roots, stems, and leaves. Additionally, we examined the persistence and conidia production of the isolates in the growing substrate. These data are vital for sustainable management practices, reducing reliance on synthetic insecticides and mineral fertilizers, and enhancing the resilience and productivity of agropastoral systems.

## 2 Materials and Methods

### 2.1 Forrage Grasses

Ten centimeter stem cuttings of *A. catharinensis*, *H. altissima*, *and C. dactylon*, were obtained from the Epagri/Cepaf forage germplasm bank in Chapecó, Santa Catarina, Brazil (27°05′24.5″ S; 52°38′09.7″ W). The cuttings were disinfected by immersion in a 2.5% (v v^−1^) sodium hypochlorite solution for 2 min, then rinsed with sterile distilled water. They were then planted in rooting tubes (2.7 cm long, 2.7 cm wide, and 12.5 cm deep) filled with autoclaved (3 cycles of 120°C and 1.1 atm for 80 min) Turfa Fertil substrate (Turfa Fértil Agro S.A., Balneário Arroio do Silva, Santa Catarina, Brazil), composed of peat, carbonized rice husk, vermiculite and fertilizers. The sterile substrate was used to minimize interference from external microorganisms during the initial isolate‐screening experiment. The cuttings were kept in a greenhouse at 26°C, with manual irrigation using sterilized distilled water as needed to maintain field capacity.

### 2.2 *Metarhizium anisopliae* Isolates

The *M. anisopliae* isolates used in this study were sourced from the Entomopathogenic Fungi Collection at Epagri/Cepaf and preserved in cryogenic tubes with 10% (v v^−1^) glycerol at −80°C. These isolates were collected from soil in pastoral regions of Santa Catarina without prior mycoinsecticide use, using larval baits of *Tenebrio molitor* L. (Coleoptera: Tenebrionidae) for isolation (insect‐trap technique) (Tables 1 and S1).

**Table 1:** Isolates of *Metarhizium anisopliae* tested for their growth‐promoting action on forage species.

Isolates	Collection site	Genbank access
CEPAF_ENT25	Canoinhas, SC	OR250326
CEPAF _ENT26	Alto da Bela Vista, SC	OR250327
CEPAF_ENT27	Campo Erê, SC	OR250328
CEPAF_ENT42	Bom Retiro, SC	OR250334
CEPAF_ENT59	Brunópolis, SC	OR250338
IBCB425	Commercial isolate (Biological Institute of São Paulo)	*—*
E9	Commercial isolate (University of São Paulo)	*—*

Identification of the tested isolates was performed using molecular analysis [15, 23]. To this purpose, PCR amplification and sequencing of nuclear intergenic loci MzFG543igs were performed, using the first pairs MzIGS3_1F (5′‐CGTGGCTCCTGACCATGGTTGC) × MzIGS3_4R (5′‐GCGGGGGAGCC GACTTGGA) and MzFG543igs_2F (5′‐TCTCTACAACCGTACCCGACA) × MzFG543igs_6R (5′‐GATAGCTCGCCTTCTGCCTTG). After the sequencing procedure, the sequences of each gene were individually checked and manually edited based on the quality of the chromatograms using the Sequencher 4.8 application (Gene Codes Corp., Ann Harbor, MI). Subsequently, the sequences were aligned using the ClustalW tool from the MEGA X application, as described in Amandio et al. [15, 23]. Consensus sequences were compared with reference sequences in GenBank (http://www.ncbi.nlm.nih.gov) (accessed on 20 July 2026), via the BLASTn program (version 2.215), and deposited under accession numbers described in Table 1. The selection of native isolates was based on their biopesticidal properties and previous evidence of growth‐promoting action in *Urochloa* spp. (former *Brachiaria*) [15]. Isolates E9 and IBCB425 were also included because they are commonly used in registered mycoinsecticides for Brazilian pasture systems [17].

### 2.3 Obtaining Conidia and Preparing Suspensions

Before the bioassays, purified *M. anisopliae* isolates were reactivated and propagated on Petri dishes (9.0 × 1.5 cm) with PDA culture medium (potato 4 g, dextrose 20 g, agar 15 g, with total dehydrated medium of 39 g L^−1^) (Difco, Becton‐Dickinson Co., Franklin Lakes, NJ, USA) and supplemented with tetracycline (0.5 g L^−1^) and streptomycin (0.5 g L^−1^). This was done in a laminar flow hood sterilized with 70% alcohol and UV light. The plates were then incubated in BOD‐type germination chambers (Model EL202, Eletrolab Indústria e Comércio de produtos para Laboratório Ltda.) for 10 days. Conidia viability was tested before use, and only isolates with viability greater than 90%, as suggested by Amandio et al. [15, 23], were selected. For this purpose, conidial viability was assessed by spreading an aliquot of each suspension onto PDA medium and incubating the plat at 25°C for 18 h. A total of 200 conidia per replicate (*n* = 5) were examined under an optical microscope, and conidia were considered germinated when the germ tube was at least equal to the conidial length.

Conidia were produced through solid‐state fermentation using parboiled rice. Conidia from various *M. anisopliae* isolates, grown in Petri dishes (see details in the previous section), were collected with a sterile spatula and immersed in sterile distilled water with Tween 80 (0.001%, v v^−1^, Sigma‐Aldrich Corp., Steinheim, Germany). A 2‐mL suspension of each isolate was inoculated into 250 mL Schott flasks containing 50 g of autoclaved parboiled rice (120°C, 1.1 atm, 25 min). After autoclaving and cooling room temperature, the parboiled rice substrate had an initial moisture content of ~58% (w w^−1^). The flasks were then incubated at 26°C with a 12‐h photoperiod in BOD‐type chambers.

After 12 days of complete substrate colonization, conidia were harvested for bioassays. Each Schott flask received 150 mL of sterile distilled water with 0.01 mL of Tween 80 and was shaken at 150 rpm for 30 min. The conidia were then filtered through sterile gauze into sterile Erlenmeyer flasks, vortexed, and serially diluted to estimate their concentration using a hemocytometer. The suspension was visualized under an optical microscope at 400× magnification. Finally, sterile distilled water was added to adjust the concentration to 1 × 10^8^ conidia mL^−1^ for the bioassays.

### 2.4 Evaluating the Growth‐Promoting Action in Seedlings of Different Forage Species

After preparing the seedlings and the rooting of the cuttings in greenhouse tubes, as previously described, 80 uniform, healthy seedlings of each species were selected. These seedlings were then placed in a phytotron set at 25 ± 2°C, 70 ± 10% relative humidity, and a 12‐h photophase.

To inoculate the plants, a conidial suspension of each *M. anisopliae* isolate (1 × 10^8^ conidia mL^−1^) was applied to five individual plants of each species using the root treatment method. The entire experiment was independently repeated once over time, with 5 additional plants used in the second experimental run, resulting in a total of 10 independent replicates per treatment (*n* = 10). Seedlings were removed from tubes, washed with sterile distilled water, and immersed in sterile Falcon tubes (50 mL) containing 30 mL of the conidial suspension for 30 min to provide standardized and direct exposure of the root system to the fungal inoculum. This procedure was based on root‐dipping approaches previously used for inoculation of plants with entomopathogenic fungi [15, 23, 39, 40, 41]. The negative control consisted of Falcon tubes containing only sterile distilled water and 0.01% Tween 80.

After inoculation, the cuttings were transplanted individually into 1050 mL polypropylene pots containing autoclaved Turfa Fertil substrate, treated at 120°C (1.1 atm) for 80 min with the aim of establishing a microbiologically simplified system reducing interference from the resident microbiota. Irrigation occurred every 2 days with 100 mL of sterile distilled water. Each was then arranged in the phytotron under conditions of 25 ± 2°C, 70 ± 10% relative humidity, and a 12‐h photophase.

Forty days after treatment and transplanting, the plants were evaluated by counting leaves and tillers and measuring the lengths of the longest stem and root, as well as the fresh and dry masses of both shoots and roots. Shoot height was measured from the base to the tip of the longest true leaf, while the roots were measured similarly. To measure dry and fresh biomass, the shoot was cut near the plant's collar, weighed on a precision analytical balance (Unibloc AUY220, Shimadzu, Philippines), placed in kraft paper bags, and dried in an oven at 60°C for 7 days. After drying, the leaves and stems were reweighed to obtain dry mass. The roots were washed to remove substrate, dried for 6 h, and then weighed. Next, they were bagged and dried in the oven (60°C for 7 days) to determine dry mass using a precision analytical balance.

### 2.5 Determining the Photosynthetic Pigment Content in Inoculated Plants

For the species that showed promising results in the growth‐promotion test, the levels of photosynthetic pigments (carotenoids and chlorophyll a and b) were measured in the central leaves of plants inoculated with different fungal isolates. Central leaves from the apical third were collected after 40 days, weighed, and stored in aluminum envelopes in an ultra‐freezer at −80°C until analysis.

The photosynthetic pigment content was determined using a spectrometric method based on Lichtenthaler [42]. Aliquots of 10 mg from each sample were macerated in liquid nitrogen, collected in septuplicate, and stored in Falcon tubes lined with aluminum foil, each containing 5 mL of 80% (v v^−1^) aqueous acetone for 24 h. The samples were then centrifuged at 5000 rpm for 20 min, and the supernatant was transferred to test tubes covered with aluminum foil to protect from the light. Absorbance readings were taken using a UV‐1800 spectrophotometer (Shimadzu Corporation, Manila, Philippines) at 470, 646.8, and 663.6 nm.

### 2.6 Endophytic Colonization by Fungal Isolates

For the species that showed promising results in the initial growth‐promotion screening, a new bioassay was conducted in order to assess the fungal colonization in plant tissues using a modified procedure from Parsa et al. [43]. To this end, two pieces of leaves, stems, and roots were collected from four randomly selected plants per treatment at 10, 20, and 40 days after fungal inoculation (independent samples). The samples were washed with sterile distilled water, disinfected in 70% (v v^−1^) alcohol for 2 min, immersed in 2.5% (v v^−1^) sodium hypochlorite for 3 min, then in 70% alcohol for 1 min, and finally rinsed in sterile distilled water. Samples were placed in sterile Petri dishes, and the outer edges were excised to remove any inner tissue that had been in contact with disinfectants, as suggested by Amandio et al. [23].

The remaining plant fragments were cut into 10 Section (2 cm per fragment) using a sterile scalpel and transferred to Petri dishes containing 8 mL of selective PDA medium with cycloheximide (0.5 g L^−1^), chloramphenicol (0.2 g L^−1^), Dodex 450 SC (*N*‐dodecyl guanidinium acetate) (0.002%), and crystal violet (0.01 g L^−1^) [22]. The plates, which held separate leaf, stem, and root fragments, were sealed and kept in a climate‐controlled chamber at 26°C with a 14‐h photoperiod for 15 days. Endophytic colonization by the fungus was confirmed by observing characteristic *Metarhizium* spp. structures (conidiophores and conidia) under a microscope at 400× magnification (Axio Scope A1, Zeiss I. Metrology, Germany), with taxonomic identification based on Driver et al. [44] and Brunner‐Mendoza et al. [45].

To evaluate the effectiveness of the cleaning process, an impression of the sterilized plant tissue was made on PDA medium after disinfection and edge removal, as described by Schultz et al. [46]. Any plates showing undesirable microorganisms led to the disposal of the corresponding sample and its replacement.

### 2.7 Persistence and Conidia Production of Isolates in the Substrate

For species that showed promising growth‐promotion results in the initial screening, a substrate samples from plants used in endophytic colonization tests were collected and preserved in sterile Eppendorf tubes at 10, 20, and 40 days after inoculation (independent samples) with *Metarhizium* sp. fungal isolates. At these evaluation intervals, 1‐g substrate samples were collected from the rhizophere zone of the plants used for colonization assays, by removing substrate directly adjacent to the root system at approximately 3 cm depth and within 3 cm of central base of each plant. These samples were suspended in sterile distilled water containing 0.01% (v v^−1^) Tween 80. This mixture of water and soil was homogenized using a vortex mixer and serially diluted (1 × 10^−1^ and 10^−2^ conidia mL^−1^), allowing for 0.5 mL of the 10^−2^ solution to be plated onto Petri dishes filled with 8 mL of selective medium as previoulsy described (Section 2.6). All plates were sealed with plastic film and kept under controlled conditions (26°C, 14‐h photoperiod) for 15 days to allow *Metarhizium* sp. colonies to establish in the culture medium. Following this incubation period, the colony‐forming units (CFU) on each plate were counted, and the species present was confirmed by examining the reproductive structures as previously described.

### 2.8 Data Analysis

A pre‐fit of the standard model was conducted on the data, with normality of residuals verified using the Shapiro‐Wilk test [47] and homogeneity of variances assessed through Bartlett's test [48]. When normality and/or homoscedasticity were not met, a Box‐Cox [49] transformation was applied. After confirming the necessary conditions, the data were analyzed using ANOVA. For treatments that showed significant differences, grouping analysis were performed using Scott‐Knott test (*p* < 0.05). All analyses were conducted using “R” version 4.0.2 [50].

## 3 Results

### 3.1 Effects of *Metarhizium anisopliae* Isolates on the Growth of Forage Plant Species

Treating seedlings of different forage species with *M. anisopliae* isolates significantly affected all vegetative growth parameters (Tables 2, 3, 4, 5). A notable interaction (*p* < 0.05) between forage species and *M. anisopliae* isolates was recorded in all assessed plant growth parameters.

**Table 2:** Fresh and dry mass of leaves of three forage species (*Axonopus catharinensis* cv. SCS 315 Catarina Gigante, and *Hemarthria altissima* cv. Florida, *Cynodon dactylon* cv. Tifton 85) inoculated with different *Metarhizium anisopliae* isolates via seedling treatment.

Treatments	Leaf fresh mass (g)				Leaf dry mass (g)			
	*C*. *dactylon*	*H*. *altissima*	*A*. *catharinensis*	Means	*C*. *dactylon*	*H*. *altissima*	*A*. *catharinensis*	Means
CEPAF_ENT25	15.15 cA	24.93 bC	30.81 aA	23.63	3.46 bA	3.99 bB	4.71 aA	4.05
CEPAF_ENT26	15.23 cA	28.01 aC	22.23 bB	21.82	3.24 bA	4.68 aB	3.68 bB	3.86
CEPAF_ENT27	11.79 cA	21.97 bC	31.94 aA	21.9	2.47 cB	4.38 bB	5.31 aA	4.05
CEPAF_ENT42	13.70 bA	25.24 aC	16.86 bC	18.6	3.00 bA	4.08 aB	2.98 bC	3.35
CEPAF_ENT59	12.63 bA	25.73 aC	21.94 aB	20.1	2.79 bA	3.97 aB	2.96 bC	3.24
E9	8.78 cB	36.64 aA	28.23 bA	24.55	2.03 bB	5.91 aA	3.9 cB	3.95
IBCB425	14.96 bA	29.75 aB	28.29 aA	24.33	3.39 bA	4.74 aB	4.23 aA	4.12
Control	8.83 cB	30.27 aB	25.69 bA	21.59	1.77 cB	4.80 aB	3.44 bB	3.37
Plant species (*F* = 212.57; *df* = 2, 216; *p* < 0.0001)					Plant species (*F* = 92.37; *df* = 2, 216; *p* < 0.0001)			
Fungal isolates (*F* = 5.03; *df* = 7, 216; *p* < 0.0001)					Fungal isolates (*F* = 5.67; *df* = 7, 216; *p* < 0.0001)			
Plant species × Fungal isolates (*F* = 7.93; *df* = 14, 216; *p* < 0.0001)					Plant species × Fungal isolates (*F* = 7.70; *df* = 14, 216; *p* < 0.0001)			

Means followed by distinct letters, uppercase in columns and lowercase in rows, indicate significant differences between isolates according to the Scott‐Knott test (*p* < 0.05).

**Table 3:** Average number of leaves and tillers of three forage species (*Axonopus catharinensis* cv. SCS 315 Catarina Gigante, and *Hemarthria altissima* cv. Florida, *Cynodon dactylon* cv. Tifton 85) inoculated with different *Metarhizium anisopliae* isolates via seedling treatment.

Treatments	Number of leaves				Number of tillers			
	*C*. *dactylon*	*H*. *altissima*	*A*. *catharinensis*	Means	*C*. *dactylon*	*H*. *altissima*	*A*. *catharinensis*	Means
CEPAF_ENT25	88.60 aA	30.80 bA	37.20 bA	52.2	12.30 aA	4.10 cA	7.00 bA	7.80
CEPAF_ENT26	75.30 aB	29.30 bA	25.50 bB	43.36	10.40 aA	4.30 bA	5.20 bA	6.63
CEPAF_ENT27	75.70 aB	25.60 cA	35.50 bA	45.60	9.80 aA	3.60 cA	6.30 bA	5.56
CEPAF_ENT42	82.00 aA	28.20 bA	23.40 bB	44.53	10.70 aA	4.00 bA	4.50 bA	6.40
CEPAF_ENT59	68.40 aB	28.30 bA	26.40 bB	41.03	8.40 aB	4.30 bA	5.30 bA	6.00
E9	54.60 aC	36.20 bA	31.00 bA	40.60	8.20 aB	5.50 bA	5.70 bA	6.46
IBCB425	83.80 aA	29.40 bA	34.00 bA	49.06	11.00 aA	4.80 bA	6.30 bA	7.36
Control	53.60 aC	32.20 bA	25.30 bB	37.03	8.30 aB	4.30 cA	6.00 bA	6.20
Plant species (*F* = 649.19; *df* = 2, 216; *p *< 0.0001)					Plant species (*F* = 180.60; *df* = 2, 216; *p* < 0.0001)			
Fungal isolates (*F* = 9.36; *df* = 7, 216; *p* < 0.0001)					Fungal isolates (*F* = 2.67; *df* = 7, 216; *p* < 0.0113)			
Plant species × Fungal isolates (*F* = 9.11; *df* = 14, 216; *p* < 0.0001)					Plant species × Fungal isolates (*F* = 2.53; *df* = 14, 216; *p* < 0.0001)			

Means followed by distinct letters, uppercase in columns and lowercase in rows, indicate significant differences between isolates according to the Scott‐Knott test (*p* < 0.05).

**Table 4:** Plant height and root length of three forage species (*Axonopus catharinensis* cv. SCS 315 Catarina Gigante, and *Hemarthria altissima* cv. Florida, *Cynodon dactylon* cv. Tifton 85) inoculated with different *Metarhizium anisopliae* isolates via seedling treatment.

Treatments	Height (cm)				Root length (cm)			
	*C. dactylon*	*H. altissima*	*A. catharinensis*	Means	*C. dactylon*	*H. altissima*	*A. catharinensis*	Means
CEPAF_ENT25	87.20 bA	115.31 aA	117.32 aA	106.61	49.52 aB	36.19 aA	44.26 aA	43.32
CEPAF_ENT26	89.65 bA	114.35 aA	76.04 cC	93.34	46.39 aB	40.93 aA	39.74 aA	42.35
CEPAF_ENT27	73.10 bB	114.39 aA	108.87 aA	99.78	51.04 aB	36.12 bA	46.51 aA	44.55
CEPAF_ENT42	77.85 bA	115.28 aA	84.74 bC	92.62	49.40 aB	31.87 bA	37.86 bA	39.71
CEPAF_ENT59	86.30 bA	120.47 aA	83.29 bC	96.68	67.07 aA	28.39 bA	32.60 bA	42.68
E9	67.65 cB	109.47 aA	94.84 bB	90.65	46.45 aB	38.08 aA	35.65 aA	40.06
IBCB425	82.58 cA	119.05 aA	97.80 bB	99.81	60.32 aA	37.35 bA	39.63 bA	45.76
Control	57.80 cB	120.63 aA	84.98 bC	87.80	48.00 aB	40.62 aA	33.79 aA	40.80
Plant species (*F* = 136.24; *df* = 2, 216; *p* < 0.0001)					Plant species (*F* = 30.75; *df* = 2, 216; *p* < 0.0001)			
Fungal isolates (*F* = 4.90; *df* = 7, 216; *p* < 0.0001)					Fungal isolates (*F* = 1.04; *df* = 7, 216; *p* < 0.4018)			
Plant species × Fungal isolates (*F* = 5.18; *df* = 14, 216; *p* < 0.0001)					Plant species × Fungal isolates (*F* = 2.72; *df* = 14, 216; *p* < 0.0001)			

Means followed by distinct letters, uppercase in columns and lowercase in rows, indicate significant differences between isolates according to the Scott‐Knott test (*p* < 0.05).

**Table 5:** Fresh and dry mass of the roots of three forage species (*Axonopus catharinensis* cv. SCS 315 Catarina Gigante, and *Hemarthria altissima* cv. Florida, *Cynodon dactylon* cv. Tifton 85) inoculated with different *Metarhizium anisopliae* isolates via seedling treatment.

Treatments	Root fresh mass (g)				Root dry mass (g)			
	*C. dactylon*	*H. altissima*	*A. catharinensis*	Means	*C. dactylon*	*H. altissima*	*A. catharinensis*	Means
CEPAF_ENT25	1.65 cA	5.12 bB	7.91 aA	4.89	1.11 aA	1.04 aB	1.23 aB	1.12
CEPAF_ENT26	1.41 cA	4.95 bB	7.59 aA	4.65	0.98 aA	1.06 aB	1.18 aB	1.07
CEPAF_ENT27	1.39 cA	4.23 bB	8.69 aA	4.77	0.95 bA	0.95 bB	1.33 aB	1.07
CEPAF_ENT42	1.10 cA	4.69 bB	6.66 aB	4.15	0.76 aA	1.09 aB	1.07 aB	0.97
CEPAF_ENT59	1.45 bA	4.56 aB	5.67 aB	3.89	1.00 bA	1.02 bB	1.86 aA	1.29
E9	1.19 bA	5.30 aB	5.48 aB	3.99	0.85 bA	1.57 aA	0.70 bC	1.04
IBCB425	1.47 cA	5.43 bB	7.57 aA	4.82	1.07 aA	1.31 aB	1.00 aB	1.12
Control	1.23 cA	7.15 aA	4.88 bB	4.42	0.81 bA	1.33 aA	0.62 bC	0.92
Plant species (*F* = 279.30; *df* = 2, 216; *p* < 0.0001)					Plant species (*F* = 9.46; *df* = 2, 216; *p* < 0.0001)			
Fungal isolates (*F* = 1.49; *df* = 7, 216; *p* < 0.1725)					Fungal isolates (*F* = 3.92; *df* = 7, 216; *p* < 0.0005)			
Plant species × Fungal isolates (*F* = 2.82; *df* = 14, 216; *p* < 0.0001)					Plant species × Fungal isolates (*F* = 7.11; *df* = 14, 216; *p* < 0.0001)			

Means followed by distinct letters, uppercase in columns and lowercase in rows, indicate significant differences between isolates according to the Scott‐Knott test (*p* < 0.05).

Increases in fresh mass of the shoots occurred in *C. dactylon* after seedling treatment with the tested *M. anisopliae* isolates, except for the commercial isolate E9, which showed no significant difference from the negative control (Table 2). Isolate E9 of *M. anisopliae* was uniquely effective in enhancing shoot fresh mass in *H. altissima*, while other isolates generally showed reductions; the isolate IBCB425 mirrored the negative control (Table 2). Conversely, none of the isolates had a positive effect on the shoot fresh mass of *A. catharinensis*, with isolates CEPAF_ENT26, CEPAF_ENT42, and CEPAF_ENT59 decreasing this parameter compared to the negative control (Table 2). Overall, the highest shoot fresh mass levels were recorded in *H. altissima*, regardless of the isolate used for seedling treatments, except for CEPAF_ENT25 and CEPAF_ENT27 (Table 2).

Most tested isolates increased leaf dry mass in *C. dactylon* (Table 2), except for plants treated with CEPAF_ENT27 and E9, which did not differ from the negative control (untreated plants; Table 2). In contrast, isolates E9 and CEPAF_ENT25, CEPAF_ENT27, and IBCB425 significantly increased leaf dry mass in *H. altissima* and *A. catharinensis*, respectively (Table 2). With exception of CEPAF_ENT25 and CEPAF_ENT27, in which *A. catharinensis* was superior, the highest leaf dry mass was recorded again in *H. altissima* (Table 2).

A similar trend was observed in the number of leaves and tillers of seedlings from three forage species treated with *M. anisopliae* isolates (Table 3). *C. dactylon* showed the most positive response, particularly with isolate CEPAF_ENT25, which increased leaf number by 65.3%. No significant differences were noted between isolates CEPAF_ENT42 and IBCB425 (Table 3). For *A. catharinensis*, the isolates CEPAF_ENT25 and CEPAF_ENT27, and the commercial isolates E9 and IBCB425, increased leaf numbers (Table 3). However, no changes were observed in *H. altissima* (Table 3). A significant response in tillers was only noted in *C. dactylon*, with CEPAF_ENT25, CEPAF_ENT26, CEPAF_ENT27, CEPAF_ENT42, and IBCB425 increasing tiller count (Table 3).

Plants of *C. dactylon* grew significantly taller than the untreated control when inoculated with all *M. anisopliae* isolates from Santa Catarina State, except for the commercial isolate E9 (Table 4). For *A. catharinensis*, CEPAF_ENT25 and CEPAF_ENT27 increased plant height by 38.05% and 28.11%, respectively (Table 4). In contrast, none of the *M. anisopliae* isolates affected the height of *H. altissima* (Table 4).

Only the isolates CEPAF_ENT59 and IBCB425 of *M. anisopliae* increased the root length of *C. dactylon* (Table 4). Conversely, the tested isolates did not affect the root lengths of *A. catharinensis* and *H. altissima* (Table 4). None of the *M. anisopliae* isolates increased the fresh or dry root mass of *C. dactylon* at 35 days after fungal inoculation (Table 5). In contrast, the fresh and dry root mass of *H. altissima* was significantly reduced (by 24.1% to 40.8%) across isolates (Table 5), except for E9 and IBCB425, which were similar to the control for dry root mass. Conversely, isolates CEPAF_ENT25, CEPAF_ENT26, and CEPAF_ENT27 increased the fresh mass of *A. catharinensis*, with all isolates except E9 increasing dry root mass (Table 5).

### 3.2 Levels of Photosynthetic Pigments in Forage Plants Treated with *Metarhizium anisopliae* Isolates

In *A. catharinensis*, isolates CEPAF_ENT25 and CEPAF_ENT26, along with CEPAF_ENT59, increased chlorophyll a, chlorophyll b, and carotenoid levels by over 20% compared to the negative control (Table 6). In contrast, no significant changes in photosynthetic pigments were observed in *C. dactylon* treated with the tested isolates (Table 6).

**Table 6:** Photosynthetic pigment contents of two forage species (*Axonopus catharinensis* cv. SCS 315 Catarina Gigante and *Cynodon dactylon* cv. Tifton 85) inoculated with different *Metarhizium anisopliae* isolates via seedling treatment.

Treatments	Chlorophyll a (mg g^−1^)		Chlorophyll b (mg g^−1^)		Carotenoids (mg g^−1^)	
	*A*. *catharinensis*	*C*. *dactylon* ^ns^	*A*. *catharinensis*	*C*. *dactylon* ^ns^	*A*. *catharinensis*	*C*. *dactylon* ^ns^
CEPAF_ENT 25	2.09 ± 0.15 a	2.00 ± 0.07	0.54 ± 0.04 a	0.57 ± 0.03	0.54 ± 0.04 a	0.51 ± 0.01
CEPAF_ENT 26	2.29 ± 0.09 a	1.78 ± 0.13	0.61 ± 0.02 a	0.44 ± 0.04	0.58 ± 0.02 a	0.47 ± 0.03
CEPAF_ENT 27	1.13 ± 0.09 c	2.28 ± 0.17	0.32 ± 0.03 b	0.53 ± 0.05	0.30 ± 0.02 b	0.59 ± 0.03
CEPAF_ENT 42	1.06 ± 0.10 c	2.00 ± 0.19	0.34 ± 0.03 b	0.47 ± 0.05	0.27 ± 0.03 b	0.52 ± 0.04
CEPAF_ENT 59	1.77 ± 0.11 b	2.08 ± 0.08	0.50 ± 0.03 a	0.52 ± 0.02	0.50 ± 0.03 a	0.54 ± 0.02
E9	1.00 ± 0.08 c	2.08 ± 0.14	0.34 ± 0.04 b	0.50 ± 0.03	0.25 ± 0.02 b	0.56 ± 0.04
IBCB 425	1.11 ± 0.11 c	2.05 ± 0.07	0.43 ± 0.03 b	0.49 ± 0.02	0.25 ± 0.03b	0.54 ± 0.02
Control	1.30 ± 0.08 c	2.01 ± 0.10	0.38 ± 0.02 b	0.46 ± 0.03	0.33 ± 0.02 b	0.53 ± 0.02
*F*	13.322	1.3753	6.0928	1.4228	24.7847	1.5756
*df*	7, 144	7, 144	7, 144	7, 144	7, 144	7, 144
*p* value	< 0.0001	0.2201	< 0.0001	0.2004	< 0.0001	0.1471

*Note:* The bioassays were conducted independently by plant species.

Means followed by distinct letters, in the columns, indicate significant differences between isolates according to the Scott‐Knott test (*p* < 0.05).

ns = not significant.

### 3.3 Endophytic Colonization and Recovery of Isolates Selected from the Substrate

The inoculated fungi were confirmed in all parts of the two selected forage species (*C. dactylon* and *A. catharinensis*). However, different frequencies of fungal recovery were observed across evaluation intervals and *M. anisopliae* isolates used for inoculation. In addition, the presence of other species of endophytic fungi was not observed in any plant's parts, as well as in the negative control.

In *A. catharinensis*, the highest endophytic colonization rates occurred 10 days after inoculation, with the fungus recovered from 41% of the roots, 38% of the stems, and 24% of the leaves (Table 7). By 20 days, these rates declined to 27% in roots, 26% in stems, and 16% in leaves. The lowest colonization rates at 40 days were 19% in roots, 12% in stems, and 6% in leaves (Table 7).

**Table 7:** Frequency of occurrence (%) of selected *Metarhizium anisopliae* isolates in samples of roots, stems, and leaves of *Axonopus catharinensis* cv. SC315 Catarina Gigante, and *Cynodon dactylon* cv. Tifton 85 and the number of colony‐forming units (CFUs) in the substrate of the pots after 10, 20, and 40 days of inoculation and transplanting of seedlings.

Treatments	10 days				20 days				40 days			
	Root (%)	Stem (%)	Leaf (%)	CFU (1 × 10^−^^2^)	Root (%)	Stem (%)	Leaf (%)	CFU (1 × 10^−2^)	Root (%)	Stem (%)	Leaf (%)	CFU (1 × 10^−2^)
*Axonopus catharinensis* cv. SC315 Catarina Gigante												
CEPAF_ENT25	32.50	22.50	25.00	325.75	15.00	35.00	22.50	223.5	22.50	12.50	10.00	154.5
CEPAF_ENT26	50.00	57.50	30.00	270.5	42.50	35.00	20.00	287	25.00	15.00	5.00	208
CEPAF_ENT27	45.00	45.00	27.50	190	27.50	20.00	12.50	177	17.50	17.50	10.00	147.75
CEPAF_ENT42	30.00	40.00	20.00	99.5	27.50	30.00	15.00	115.25	22.50	12.50	7.50	87.5
CEPAF_ENT59	60.00	50.00	32.50	100.5	42.50	35.00	25.00	153.75	27.50	20.00	2.50	148.25
E9	27.50	17.50	12.50	72.25	17.50	10.00	7.50	91.75	7.50	2.50	5.00	69.75
IBCB425	45.00	35.00	20.00	88	15.00	17.50	7.50	10.25	7.50	5.00	0.00	20.75
Control	0.00	0.00	0.00	0	0,00	0.00	0.00	0	0.00	0.00	0.00	0
*Cynodon dactylon* cv. Tifton 85												
CEPAF_ENT25	35.00	67.50	32.50	140.25	25.00	10.00	5.00	136.75	0.00	0.00	0.00	128.5
CEPAF_ENT26	35.00	87.50	45.00	167	25.00	45.00	2.50	122.25	20.00	20.00	10.00	61.25
CEPAF_ENT27	22.50	67.50	20.00	61	35.00	40.00	15.00	35	7.50	5.00	0.00	58.5
CEPAF_ENT42	27.50	37.50	15.00	97.75	2.50	15.00	2.50	81	22.50	12.50	0.00	24.25
CEPAF_ENT59	37.50	67.50	35.00	171.75	77.50	87.50	27.50	182	12.50	10.00	2.50	118.5
E9	2.50	37.50	10.00	60.5	30.00	27.50	7.50	62.75	15.00	5.00	0.00	13
IBCB425	23	15.00	10.00	32.75	12.50	12.50	7.50	10.5	15.00	5.00	0.00	60.75
Control	0	0	0	0	0	0	0	0	0.00	0.00	0.00	0

The endophytic colonization rate of *A. catharinensis* 10 days after inoculation showed CEPAF_ENT59 as the most notable isolate, recovered from 47.50% of the plated plant tissues, followed closely by CEPAF_ENT26 (45.83%). Other isolates included CEPAF_ENT27 (39.17%), IBCB425 (33.33%), CEPAF_ENT42 (30%), CEPAF_ENT25 (26.67%), and the commercial isolate E9 at 19.17% (Table 7). CEPAF_ENT59 and CEPAF_ENT26 maintained the highest colonization rates throughout the evaluation, with 34.17% and 32.50% at 20 days, and 16.67% and 15% at 40 days, respectively. Overall, CEPAF_ENT26 (32.50%) had the highest colonization across all periods, followed by CEPAF_ENT42 (29.72%), CEPAF_ENT59 and CEPAF_ENT25 (both with 29.44%), CEPAF_ENT27 (25%), IBCB425 (18.33%), and E9 (9.17%) (Table 7).

The colony‐forming unit (CFU) counts from plates inoculated with substrate solutions from seedlings treated with fungal isolates decreased over time, reflecting a decline in viable conidia. After 10 days, the average CFU count was 327.38 × 10^2^ CFU mL^−1^. By 20 days, conidia in the substrate were reduced by 7.68%, with the lowest count at 40 days, averaging 239 × 10^2^ CFU mL^−1^, representing a 27% decrease from the initial count (Table 7). Notably, the highest CFU counts were observed for isolates CEPAF_ENT26 and CEPAF_ENT25 (Table 7).

Similar to *A. catharinensis*, the highest endophytic colonization in *C. dactylon* occurred 10 days post‐inoculation, with stems at 54.43%, roots at 31%, and leaves at 26.07% (Table 7). This pattern continued at 20 days, with stems at 33.93%, roots at 29.64%, and leaves at 9.64%. By 40 days, colonization rates declined, with roots at 4.31%, stems at 3.57%, and leaves at 1.43% (Table 7). Among the isolates tested, CEPAF_ENT26 had the highest rate at 10 days (55.83%), followed by CEPAF_ENT59 (46.67%), CEPAF_ENT25 (45%), and CEPAF_ENT27 (36.67%). At 20 days, CEPAF_ENT59 reached 64.17%, and CEPAF_ENT27 reached 30%, while all other isolates were below 25%, showing the presence of the inoculated fungi. CEPAF_ENT25 was the only isolate above 5% at 40 days, showing 16.67% (Table 7).

The CFU count in the substrate of *C. dactylon* plants inoculated with *M. anisopliae* isolates gradually decreased over time (Table 7). CEPAF_ENT59 had the highest count at 314.4 × 10^2^ CFU mL^−1^, followed by CEPAF_ENT25 at 270.34 × 10^2^ CFU mL^−1^, and CEPAF_ENT26 at 233.66 × 10^2^ CFU mL^−1^. Other isolates ranged from 134 × 10^2^ CFU mL^−1^ (CEPAF_ENT42) to 69.34 × 10^2^ CFU mL^−1^ (IBCB 425) (Table 7).

## 4 Discussion

Endophytic colonization of important agricultural plants by entomopathogenic fungi can boost growth and stress resilience [15, 16, 38, 51]. Although *M. anisopliae* is widely used for its pesticidal properties [17, 52], this study is the first to show that native isolates can be applied to seedlings of *A. catharinensis*, *H. altissima*, and *C. dactylon*, which are key forage species in southern Brazil [7, 8]. This inoculation method effectively promoted endophytic colonization in seedlings, with results varying by plant species and the specific isolate used. Propagules of this fungus were detected in all parts of the analyzed plants, including roots, stems, and leaves. Supporting findings from previous research, seed and seedling inoculation techniques have been employed to establish a protocooperative association between entomopathogenic fungi and plants of agricultural significance [22, 23, 24]. However, these fungi are more frequently found inhabiting the roots of such plant species [15, 24, 53].

The plant‐endophyte interactions, particularly those associated with plant growth promotion, can be influenced by several biological and experimental factors. Environmental conditions, plant physiological status, inoculum quality, and greenhouse batch effects may affect fungal establishment, plant responses, and the magnitude of growth‐promoting effects should be interpreted within this context [38, 54, 55, 56]. In this context, the impact of the analyzed species on plant growth varied based on the isolates used and the forage species examined. For *C. dactylon* and *A. catharinensis* seedlings, specific isolates led to significant increases in the dry and fresh mass of leaves and roots, as well as greater leaf counts and plant heights. Conversely, similar benefits were generally not observed for *H. altissima*, where some isolates even caused reductions in developmental parameters with higher inoculation levels. This comparatively limited growth response of *H. altissima* may reflect a less favorable compatibility between this plant host and the *Metarhizium* isolates tested. Plant responses to entomopathogenic fungi are strongly context‐dependent and can vary according to host genotype, fungal strain, physiological status, and the balance between the metabolic costs and benefits associated with fungal establishment [31, 38, 57]. In a less compatible host, fungal establishment may not translate into measurable growth promotion and may instead impose transient metabolic costs associated with defense activation, competition for nutrients, or maintenance of the plant‐fungus interaction [30, 58, 59]. In the present study, the absence of consisted increases in plant height and root length, together with reductions in root biomass for several isolates, suggests that *H. altissima* may derive fewer growth‐related benefits from the tested isolates than *A. catharinensi*s and *C. dactylon*. These results suggest that *M. anisopliae* displays a species‐specific ability to colonize forage grasses, ranging from antagonism to mutualism, depending on the fungal isolate and environmental conditions [51, 60]. This temporary imbalance during the early growth phase may lead to cumulative costs that are not resolved by the end of the forage's growth cycle, which could explain the negative results observed in *H. altissima* [61].

Most studies on entomopathogenic fungi and cultivated plants report positive or neutral outcomes [24, 62]; however, some indicate a decrease in productivity post‐inoculation [16]. For example, Garrido‐Jurado et al. [63] found that wheat plants inoculated with *Metarhizium brunneum* (Metsc.). Sorokin (Hypocreales: Clavicipitaceae) showed a significant reduction in leaf count and root dry matter. Inoculation of *Beauveria bassiana* (Bals.) (Ascomycota: Hypocreales) isolates via seed treatment has been shown to inhibit maize plant growth [60]. This growth reduction results from competition for nutrients between the fungal isolates and the seedlings [51], indicating that the fungus can inhibit plant growth in certain situations [16]. Thus, studies that select new isolates and evaluate their interactions with various forage plant species are essential for developing innovative products from multifunctional entomopathogenic fungi [21, 62].

The response to inoculation varied among the tested grasses, indicating isolate specificity. The isolates CEPAF_ENT25 and CEPAF_ENT27 reduced growth in *H. altissima* but increased growth in *A. catharinensis* and *C. dactylon*. In contrast, the commercial isolate IBCB425 produced stable, intermediate results across all species, indicating its adaptability. The biostimulant effect of fungi arises from direct actions, such as increased production of phytohormones and photosynthetic compounds, as well as indirect actions, including induction of systemic resistance against pests and pathogens [64, 65, 66]. In this study, we found that isolates CEPAF_ENT25 and CEPAF_ENT26 significantly boosted chlorophyll a, b, and carotenoid production in inoculated *A. catharinensis* plants. González‐Pérez et al. [30] also reported increased total chlorophyll in *Arabidopsis* plants with *M. anisopliae*. Furthermore, Moustaka et al. [67] noted that *M. brunneum* enhanced photosynthetic efficiency in plants after herbivory. Previous studies have shown that mycorrhizal colonization enhances carbon production through improved photosynthesis. However, this increase in carbon does not always lead to biomass gains for plants; instead, it can promote the development of root‐associated microorganisms [24, 68]. Thus, the rise in photosynthetic compounds observed in *A. catharinensis* likely results from the activity of inoculated fungal isolates aiming to meet their own carbon needs, a topic that requires further exploration. Future investigations should also address the potential effects of fungal colonization on forage chemical composition and food safety for dairy livestock. Species of *Metarhizium* are known to produce biologically active secondary metabolites (mycotoxins), among which destruxins are particularly relevant [69, 70]. Although their presence in colonized forage does not necessarily imply a toxicological risk to animals [71], scientific evidence regarding their safety in animal feed remains limited, warranting further investigation.

Isolates of *M. anisopliae* were recovered from all parts of the inoculated plants, most frequently from the roots, likely due to the inoculation method. Fungal isolation was also observed in stem and leaf tissues, particularly 10 days after inoculation, indicating endophytic activity. However, the colonization rate significantly decreased over time, consistent with findings from other studies on crops exposed to entomopathogenic fungi [24, 43]. Jaber and Ownley [62] suggest that the persistence of endophytic colonization may be improved with periodic applications of the microbial agent using various inoculation techniques, which warrants further research in future field studies. An additional aspect to be consider is that the use of an autoclaved substrate provided a microbiologically simplified and controlled system, allowing the effects of the inoculated *Metharhizium* isolates to be evaluated with reduced interference from the resident soil microbiota. This approach is particularly useful for initial screening and for comparing fungal isolates under standardized conditions. Under natural pasture conditions, however, *Metharhizium* interacts with a more complex rhizosphere environment, where microbial interations, plant genotype, root exudates, and the resident fungal community may influence fungal establishment and persistence [36, 37]. Previous studies have shown that the occurrence of *Metharhizium* associated with crop roots can reflect its diversity and abundance in surrounding sois [36], while also demonstrating its competitive capacity and ability to establish plant associations in the presence of native soil microbiota [35, 37, 72]. Therefore, the results presented here provide a useful basis for subsequent validation under non‐sterile soil and field conditions, as also demonstrated in other recent studies [15, 23, 38, 59, 73].

This study demonstrated that immersing roots in a conidia suspension effectively established endophytes in forage grasses, with persistence for up to 40 days. Although the mechanisms underlying this decline were not directly evaluated, previous studies have shown that the survival and persistence of *Metarhizium* propagules in soil and growing substrate are strongly influenced by temperature, moisture, water availability, and other physicochemical characteristics of substrate [74, 75, 76]. However, Canassa et al. [21] showed that *M. robertsii* and *B. bassiana* can persist for up to 6 months in strawberry plant substrates after root treatment.

Although root dipping maximizes contact between fungal propagules and the root system [43], its practical applicability for large‐scale pasture establishment is restricted to species commonly propagated by seedlings, as is the case for the three forage species evaluated in this study. More scalable delivery strategies, including seed treatment or coating, soil‐directed application, in furrow delivery, and granular formulation, should therefore be considered in further studies. Seed inoculation with *Metharhizium* spp. has successfully promoted endophytic establishment and plant growth in different crops including grass species [24, 77], whereas soil‐ and granule‐based formulation can provide alternative approaches for delivering viable propagules directly to the rhizosphere [78, 79, 80]. Nevertheless, further field‐scale studies are needed to determine suitable inoculum doses, formulation types, persistence, operational costs, and cost‐benefit relationships before these approaches can be recommend for commercial pasture establishment.

In conclusion, the native isolates CEPAF_ENT25 and CEPAF_ENT27, along with the commercial isolate IBCB425 of *M. anisopliae*, successfully established endophytism in *A. catharinensis* and *C. dactylon*, leading to increased biomass. Exploring new entomopathogenic fungal isolates tailored to specific edaphoclimatic conditions and forage species could foster innovative products. This research is vital for enhancing integrated pest management (IPM) strategies in pastures, where *Metarhizium* spp. conidia are commonly used to control spittlebug populations and improve forage plant nutrition.

## Author Contributions

**Dylan Thomas Telles Amandio:** conceptualization, investigation, writing – original draft, methodology, validation. **Alex Sandro Poltronieri:** conceptualization, writing – original draft, methodology, validation, writing – review and editing. **Michele Trombin de Souza:** writing – original draft, visualization, writing–review and editing. **Leandro do Prado Ribeiro:** conceptualization, investigation, funding acquisition, writing – original draft, methodology, validation, visualization, writing – review and editing, formal analysis.

## Conflicts of Interest

The authors declare no conflicts of interest.

## Supporting information


Supporting File

## Data Availability

The data that support the findings of this study are available from the corresponding author upon reasonable request.
