# Supplementary material for: Beyond Biocontrol: Species‐Dependent Growth Promotion of Forage Grasses by Metarhizium anisopliae Isolates
Source: J Basic Microbiol. 2026 Oct 27;66(10):e70211. doi: 10.1002/jobm.70211 (PMC13711243; doi:10.1002/jobm.70211)
Supplement: Supplementary file 1 [file JOBM-66-e70211-s001.docx]

**Supporting Information**

**Table S1** Performance of *M. anisopliae* isolates on the growth parameters of *Axonopus catharinensis* cv. SCS 315 Catarina Gigante, *Hemarthria altissima* cv. Florida, and *Cynodon dactylon* cv. Tifton 85

| Leaf fresh mas | | | Leaf number | | | Leaf dry mass | | | Tillers | | | Height | | | Root length | | | Root fresh mass | | | Root dry mass | | | |
| --- | --- | --- | --- | --- | --- | --- | --- | --- | --- | --- | --- | --- | --- | --- | --- | --- | --- | --- | --- | --- | --- | --- | --- | --- |
| **A¹** | **H²** | **C³** | **A** | **H** | **C** | **A** | **H** | **C** | **A** | **H** | **C** | **A** | **H** | **C** | **A** | **H** | **C** | **A** | **H** | **C** | **A** | **H** | **C** |  |
| 27 | E9 | 26 | 25 | ns | 25 | 27 | E9 | 25 | ns | ns | 25 | 25 | ns | 26 | ns | ns | 59 | 27 | C | ns | 59 | E9 | ns |  |
| 25 | C | 25 | 27 |  | IBCB | 25 | C | IBCB |  |  | IBCB | 27 |  | 25 |  |  | IBCB | 25 | IBCB |  | 27 | C |  |  |
| IBCB | IBCB | IBCB | IBCB |  | 42 | IBCB | IBCB | 26 |  |  | 42 | IBCB |  | 59 |  |  | 27 | 26 | E9 |  | 25 | IBCB |  |  |
| E9 | 26 | 42 | E9 |  | 27 | E9 | 26 | 42 |  |  | 26 | E9 |  | IBCB |  |  | 25 | IBCB | 25 |  | 26 | 42 |  |  |
| C | 59 | 59 | 59 |  | 26 | 26 | 27 | 59 |  |  | 27 | C |  | 42 |  |  | 42 | 42 | 26 |  | 42 | 26 |  |  |
| 26 | 42 | 27 | 26 |  | 59 | C | 42 | 27 |  |  | 59 | 42 |  | 27 |  |  | C | 59 | 42 |  | IBCB | 25 |  |  |
| 59 | 25 | C | C |  | E9 | 42 | 25 | E9 |  |  | C | 59 |  | E9 |  |  | E9 | E9 | 59 |  | E9 | 59 |  |  |
| 42 | 27 | E9 | 42 |  | C | 59 | 27 | C |  |  | E9 | 26 |  | C |  |  | 26 | C | 27 |  | C | 27 |  |  |

**A¹**= *Axonopus catharinensis* cv. SCS 315 Catarina Gigante; **H²**=*Hemarthria altissima* cv. Florida; **C³**=*Cynodon dactylon* cv. Tifton 85.

Note: 25 = Isolate CEPAF_ENT25; 26 = Isolate CEPAF_ENT26; 27 = Isolate CEPAF_ENT27; 42 = Isolate CEPAF_ENT42; 59 = Isolate CEPAF_ENT59; IBCB = Isolate IBCB425; C = Control.

ns: not significant
